# Patients’ knowledge about their involvement in clinical trials. A non-randomized controlled trial

**DOI:** 10.3389/fmed.2022.993086

**Published:** 2022-09-20

**Authors:** Pablo Juan-Salvadores, Marcela Sánchez Michel Gómez, Víctor Alfonso Jiménez Díaz, Cristina Martínez Reglero, Andrés Iñiguez Romo

**Affiliations:** ^1^Cardiovascular Research Unit, Cardiology Department, Hospital Álvaro Cunqueiro, University Hospital of Vigo, Vigo, Spain; ^2^Cardiovascular Research Group, Galicia Sur Health Research Institute (IIS Galicia Sur), SERGAS-UVIGO, Vigo, Spain; ^3^Interventional Cardiology Unit, Cardiology Department, Hospital Álvaro Cunqueiro, University Hospital of Vigo, Vigo, Spain; ^4^Methodology and Statistics Unit, Galicia Sur Health Research Institute (IIS Galicia Sur), SERGAS-UVIGO, Vigo, Spain; ^5^Cardiology Department, Hospital Álvaro Cunqueiro, University Hospital of Vigo, Vigo, Spain

**Keywords:** bioethics, informed consent, educational research, clinical trials, communication in research, trial participants, evaluation research

## Abstract

**Background:**

Nowadays, good clinical practice should be established in human research. Patient’s rights and autonomy must be respected above the interest of the researcher, making mandatory to raise patient’s awareness on the implications of participating in a clinical study. Contrary to popular belief, this is not always the case. This means that, after signing the informed consent form, some patients have difficulties understanding their responsibilities as participants.

**Materials and methods:**

This study is a prospective, multicenter, non-randomized controlled trial comparative survey conducted on patients enrolled in a clinical trial to evaluate and improve their understanding after an educational intervention was applied to the research staff.

**Results:**

Females were underrepresented in the clinical trials performed in this study, 21.5%. Most of the participants had a low educational level (74.4%). Around 5 and 10% of the research participants were not aware they were part of a clinical study, and more 24% just trusted in the medical decision to be enrolled. After the interventional education, the following items: “given time and resolution of the patient doubts” (*p*-value = 0.003), “enough written information” (*p*-value = 0.006), “explanation of the risks of participating in the study,” (*p*-value = 0.047) and understanding of the information provided to them showed an improvement regarding the study in which they were participating.

**Conclusion:**

The research participants understanding of their involvement in clinical trials is limited. An educational intervention on the research team can improve the process of empowerment and transit of information.

## Introduction

Biomedical research is an activity designed to generate, develop, and contribute to evidence-based knowledge. Much of the progress of medicine is based on biomedical research. Year after year we are seeing an increase in the number of clinical trials and with the COVID-19 pandemic it has been an unprecedented explosion (1). As a result, a series of requirements are considered. Clinical research ethics is guided by the classic bioethics model composed of main components unified in the International Conference on Harmonization Guidance on Good Clinical Practices (ICH-GCP) (2).

In the context of clinical research, respect for persons is the basis for voluntary participation. The informed consent form (ICF) is the maximum expression of this bioethical principle (3). For a patient to experience all-embracing autonomy at the time of inclusion, the participant should have full disclosure, comprehension of the information provided and willingness to participate (4, 5). Patient’s rights and autonomy must be respected above the interest of the researcher and the pharmaceutical industry. Even though Ethics Committees should take the time to review patient information sheets of clinical trials before approval and conduct (6–8), their resources are often too limited to evaluate the consent process and actual patient comprehension (9). Contrary to popular belief, it is documented that it is still difficult for some patients to understand their rights and responsibilities as clinical trial participants (10–12), violating basic ethical and legal principles and resulting in an overall endangering of the validity of trial results.

A literature review of previous studies has evidenced that formal steps for obtaining clinical trial informed consent are usually carried out. However, regarding participants ì knowledge of key aspects of clinical trials, it is reported that 98% recalled having signed the ICF and 87% of them felt there were well informed. However, 26% reported to have signed the consent form without actually reading the ICF (10). Research staff plays an essential role in the physician-patient relationship (12–14). Improvement in patient understanding of trial methods and patient safety could enhance interest and clinical trial recruitment (9). Upgrading the understanding by bioresearch staff of the design, conduct and ethics in research, we can improve the quality and integrity of clinical research (4). Hence, patient’s understanding of the ICF has not received the attention it deserves (11).

Our main objectives are to determine the patient’s understanding of what the clinical trial entails and to assess the impact of an educational intervention conducted on the clinical research staff.

## Materials and methods

A multicenter, prospective non-randomized controlled trial comparative survey conducted before and after an educational intervention conducted in four medical departments (Hemodynamics, Electrophysiology, Oncology, Internal Medicine) that had more clinical interventional trials ongoing at the Hospital Meixoeiro and Hospital Xeral Cíes, which are part of the Social Security Health System of Vigo, in northwestern Spain.

**FIGURE 1:**
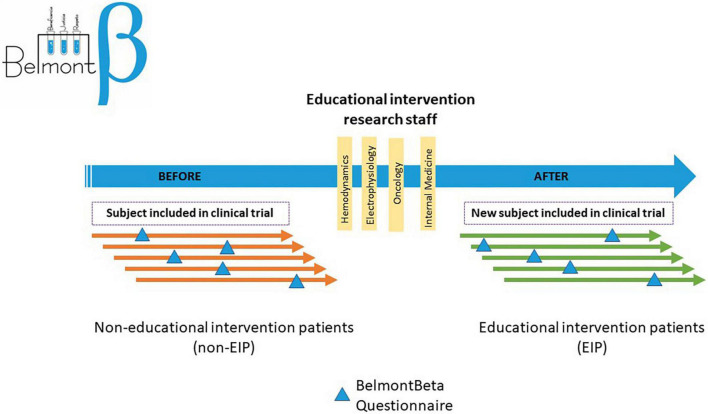
Flow chart diagram of the study.

The study consisted of two evaluations; the first evaluation was conducted in patients included in clinical trials by the staff before the designed educational intervention was implemented and a second comparative evaluation on a different kind of trial patient population by the same staff after the intervention (Figure 1).

### Inclusion/exclusion criteria

Inclusion criteria for the non-educational intervention patient (non-EIP) group: all patients over 18 years old participating in ongoing clinical trials.

Exclusion criteria for non-EIP group: Cognitive or physical inability to complete the questionnaire, for more information see survey design. Answering less than 90% of the questions.

Inclusion criteria for the educational intervention patient (EIP) group: all patients over 18 years old participating in ongoing clinical trials after the educational intervention on research staff.

Exclusion criteria for the EIP group: Cognitive or physical inability to complete the questionnaire. Answering less than 90% of the questions. Patients who had been evaluated in the non-EIP group. Patients should not be included at the same date of inclusion in their active clinical trial.

### Sample size

Previous studies have reported that the percentage of understanding of the information received by the non-EIP group is about 50% (9). Thus, to obtain a power of 80% to detect differences in the contrast of the null hypothesis through the Chi-square test, with a significance level of 5%, we assume that the percentage of the EIP group is 70%. According to this, 93 research participants per group should be included in the study. Moreover, it is estimated that the percentage of uncompleted questionnaires will be 5%, 97 non-educational and educational intervention research participants should be recruited.

### Data collection

Data collection for the questionnaires for the non-EIP/EIP groups was obtained by voluntary inclusion of all the patients included in any interventional trial of the clinical departments involved, for 12 months from September 2014 to February 2015 (first inclusion period) and from June 2015 to September 2017 (second inclusion period). To ensure confidentiality and willingness, patients were not asked for any personal information and were instructed at the end of questionnaire to tear and hand the Physician Annex to their clinical research staff. Both surveys should be placed in a specific container located at a neutral area (waiting room) of the clinical departments.

### Educational intervention

As all of the staff included in the educational intervention already had experience in clinical research and had a ICH GCP certificate, the educational session included a 45-min session approved and certified by the Health Department of Galicia (*Xunta de Galicia, Consellería de Sanidade*) with approved #11-0008-09/0023-A worth 0.2 credits on continuous education, that took place in each of the participating medical departments with the support of visual and written information delivered by the principal investigator.

The educational session included a review of basic concepts of the history and development of bioethics and modern clinical trials, ICH GCP guidelines and fundamental requirements relating to subject protection, physician-patient relationship, essential verbal, and written information on the ICF, and patient involvement in clinical trials. There was also a discussion of the most significant results of the non-EIP group questionnaires previously evaluated. And it ended with a brief seminar discussion with practical exercises of different clinical settings. These seminars concluded with the following recommendations: First, make sure the participants are properly informed by taking the time necessary. Second, read the patient information sheet together, combining the written information with verbal clarification in a language that is not scientific. The third step is to give them ample time to decide whether or not to participate.

### Survey design

The questionnaire (Supplementary material) was designed specifically for this study by a multidisciplinary group of people with previous experience in questionnaire design (15). The survey was based on the Integrated Addendum to ICH E6 (R1): Guideline for Good Clinical Practice as well as on the Spain and European Union legal requirements. The questionnaire comprises seven pages containing two annexes, a patient questionnaire (Annex I) and an investigator questionnaire (Annex II).

The patient questionnaire comprised 38 items, seven of which were designed for gathering social-demographic data and 31 for understanding patient involvement in the clinical trial. To evaluate the knowledge of patient involvement, the questions were categorized into three dimensions: 15 questions were related to patient comprehension, 12 for given information and 4 for patient willingness (voluntariness). The physician questionnaire was designed to compare patient answers to physician answers and comprised 12 items, six of which were for general aspects of the trial and six specifics of the trial design.

Grammar and syntax were reviewed and corrected by two specialized members of the Research Ethics Committee of Galicia to assess the validity of the survey and applied in a pilot rehearsal with ten non-clinical staff members of the Cardiology Department.

### Statistical analysis variables

A descriptive analysis was made with the results obtained from the patient questionnaires. Socio-demographic variables such as age, sex, educational level, marital, and employment status are described as independent variables and level of the knowledge of the involvement of the patient included in a clinical trial was considered as the dependent variable. Quantitative variables were presented as mean, 95% confidence intervals and standard deviation. On the other hand, the qualitative variables were given as frequencies and percentages. The Chi-square test was used for univariate analysis between the main study qualitative variables (non-EIP/EIP group questionnaires). The Student’s *t*-test was used to compare any numeric variables (age, etc.). These statistical analyses were performed using SPSS v19.

The analysis of the agreement of the answers of the patient and the investigator was performed using the Kappa index in each group. The significance of the educational intervention for consistency was carried out with the homogeneity of the Kappa test. The Epidat v3.1 software was used for this analysis.

### Ethical and legal aspects

This study has been conducted according to the applicable ethical and legal laws, the Declaration of Helsinki, and GCP on humans. The study has been approved by the research Ethics Committee Recognized by *Consellería de Sanidade*, with registration number: 2014/329.

## Results

During the trial period 258 questionnaires were completed, 223 of which were included in the trial and 35 were excluded for meeting exclusion criteria. There were 153 questionnaires in the non-EIP group and 70 in the EIP group.

In both groups most of the participants were male, 78.5% were in the sixth decade of life, and more than 70% of participants had a primary education level or lower. There were no baseline statistical differences between the groups evaluated. The rest of the socio-demographic variables are described in Table 1.

**TABLE 1:** Characteristics of the sample.

	Population (*n* = 223)	Groups		*P*-value
		Non-EIP (*n* = 153)	EIP (*n* = 70)	
**Age (years)**	61.46 ± 11	60.78 ± 11	63.42 ± 12	0.148
**Sex**				
Male	175 (78.5%)	118 (77.1%)	57 (81.4%)	0.599
Female	48 (21.5%)	35 (21.9%)	13 (18.6%)	
**Marital status**				
Single	19 (8.5%)	12 (7.8%)	7 (10.0%)	0.483
Married or living with a couple	174 (78.0%)	118 (77.1%)	56 (80.0%)	
Divorced/separated	10 (4.5%)	9 (5.9%)	1 (1.4%)	
Widow	20 (9.0%)	14 (9.2%)	6 (8.6%)	
**Level of education**				
Incomplete primary education	85 (38.1%)	54 (35.3%)	31 (44.3%)	0.611
Primary education	72 (32.3%)	50 (32.7%)	22 (31.4%)	
Medium education	39 (17.5%)	29 (19.0%)	10 (14.3%)	
Higher education	25 (11.2%)	18 (11.8%)	7 (10.0%)	
DK/NA/REF	2 (0.9%)	2 (1.3%)	0 (0%)	
**Employment status**				
Employed	69 (30.9%)	50 (32.7%)	19 (27.1%)	0.594
Unemployed	21 (9.4%)	14 (9.2%)	7 (10.0%)	
Housework	12 (5.4%)	8 (5.2%)	4 (5.7%)	
Retired or pensioner	120 (53.8%)	81 (52.9%)	39 (55.7%)	
DK/NA/REF	1 (0.7%)	0 (0%)	1 (1.4%)	
**Main language**				
Spanish	132 (59.2%)	94 (61.4%)	38 (54.3%)	0.409
Galician	71 (31.8%)	44 (28.8%)	27 (38.6%)	
Others	2 (0.9%)	1 (0.7%)	1 (1.4%)	
DK/NA/REF	18 (8.1%)	14 (9.2%)	4 (5.7%)	

DK, does not know; N/A, not available; REF, refusal; EI, educational intervention; Non-EIP, non-educational intervention patient.

Regarding the Investigator questionnaire (Annex 2), most of the patients included in each of the evaluations (non-EIP/EIP groups) were actively participating in multicenter studies, 93.5/91.4%, and in international 92.2/81.4%. The description of the clinical trials where patients were actively involved is given in Table 2.

**TABLE 2:** Annex II–Research staff answers.

	Population (*n* = 223)	Groups		*P*-value
		Non-EIP (*n* = 153)	EIP (*n* = 70)	
**Study objective**				
Drug	112 (50.2%)	71 (46.4%)	41 (58.6%)	0.009
Device	76 (34.1%)	55 (35.9%)	21 (30.0%)	
Both	16 (7.2%)	16 (10.6%)	0 (0%)	
None	55 (35.9%)	2 (1.3%)	5 (7.1%)	
DK/NA/REF	2 (0.9%)	2 (1.3%)	0 (0%)	
**Clinical trial phase**				
Phase II	3 (1.3%)	0 (0%)	3 (4.3%)	0.019
Phase III	96 (43.0%)	72 (47.1%)	24 (34.3%)	
Phase IV	29 (13.0%)	16 (10.5%)	13 (18.6%)	
DK/NA/REF	2 (0.9%)	2 (1.3%)	0 (0%)	
**Extraordinary tests**				
Yes	172 (77.1%)	109 (71.2%)	63 (90.0%)	0.002
Not	39 (17.5%)	36 (23.5%)	3 (4.3%)	
**Methodology**				
Randomized	191 (90.1%)	129 (89.0%)	62 (92.5%)	0.481
Non-randomized	18 (8.5%)	13 (9.0%)	5 (7.5%)	
DK/NA/REF	3 (1.4%)	3 (2.1%)	0	
**Methodology II**				
Single-blind	50 (22.4%)	40 (26.1%)	10 (14.3%)	0.175
Double-blind	92 (41.3%)	58 (37.9%)	34 (48.6%)	
Unblinded	68 (30.5%)	45 (29.4%)	23 (32.9%)	
DK/NA/REF	2 (0.9%)	2 (1.3%)	0 (0%)	
**Placebo use**				
Yes	88 (41.5%)	57 (39.3%)	31 (46.3%)	0.317
Not	122 (57.5%)	86 (59.3%)	36 (53.7%)	
DK/NA/REF	2 (0.9%)	2 (1.3%)	0 (0%)	
**Number of ICF pages**	13.70 ± 5.67	14.14 ± 5.90	12.76 ± 5.76	0.100
**Number of extraordinary visits**	10.33 ± 10.89	9.77 ± 11.22	11.71 ± 9.75	0.247
**Time between survey and inclusion (months)**	9.27 ± 9.38	12.17 ± 9.95	2.94 ± 2.40	0.000

DK, does not know; N/A, not available; REF, refusal; EI, educational intervention; Non-EIP, non-educational intervention patient.

The results on willingness are shown in Table 3. Regarding the questions “Given Information,” most of patients in the non-EIP group were invited to participate in the clinical trial at the time of their surgical procedure 37.9%, compared to the EIP group that shows a change in the location of the doctor’s office 77.1%. The rest of the results are described in Table 4.

**TABLE 3:** Willingness questions.

	Yes* Non-EIP (*n* = 153)	Yes* EIP (*n* = 70)	*P*-value
Have you read the whole informed consent form before you agreed to take part in the study?	107 (69.9%)	55 (78.6%)	0.113
Do you think you have had enough time to think through and give an answer for your participation in the study?	115 (75.2%)	64 (91.4%)	0.003
Have you been offered the possibility to take it home and discuss it with any relatives or friends before signing it?	61 (39.9%)	45 (64.3%)	0.000
Can you withdraw your consent and leave the study whenever you want?	105 (68.6%)	56 (80.0%)	0.149
Would you mind participating in a future study if you were asked to?	139 (90.8%)	66 (94.3%)	0.436

*Yes, or correct answer.

EI, educational intervention; Non-EIP, non-educational intervention patient.

**TABLE 4:** Given information.

	Yes* Non-EIP (*n* = 153)	Yes* EIP (*n* = 70)	*P*-value
Do you know the disease for which you have been invited to take part in the study?	141 (92.2%)	67 (95.7%)	0.667
Have you been informed if you could get any benefits from your participation?	82 (53.6%)	39 (55.7%)	0.522
Are you aware or has someone explained to you the risks of participating in the study?	89 (58.2%)	50 (71.4%)	0.047
Do you think the written information given to you was enough?	104 (68.0%)	61 (87.1%)	0.006
Have you got any copy of the informed consent form that you had to sign?	106 (69.3%)	56 (80.0%)	0.143
If you received a copy of the informed consent form that you have signed, is this copy signed by the investigator that invited you to participate?	75 (49.0%)	46 (65.7%)	0.137
Do you think the information was easy to understand?	93 (60.8%)	54 (77.1%)	0.017
Have you got the chance to discuss with the investigator the contents and any doubts you had?	123 (80.4%)	64 (91.4%)	0.048
Would you like to see the results obtained from the research you are participating in?	48 (31.4%)	19 (27.1%)	0.509

*Yes, or correct answer.

EI, educational intervention; Non-EIP, non-educational intervention patient.

Regarding the comprehension questions, we found that more than a half of the patients in both groups answered they knew the clinical trial was related to the administration of a drug 58.2/60.0% and more than two-thirds reported complete or good satisfaction in the process of clarification of their clinical trial doubts 74.1/85.8%. On behalf of the reasons leading the investigator to invite them to the trial, 24.2/24.3% just trusted the medical decision for enrollment. Also, some of the patients were not acquainted with the idea they had extraordinary clinical visits 22.9/27.1%, or extra tests 28.1/21.4, and 55.6/47.1% did not know if a placebo was used as part of the trial. The rest of the results are shown in Table 5.

**TABLE 5:** Comprehension answers.

	Yes* Non-EIP (*n* = 153)	Yes* EIP (*n* = 70)	*P*-value
Do you think you have understood the aim of the study you are participating in?	124 (81.0%)	63 (90.0%)	0.136
Do you know what it means that your participation is blind?	60 (39.2%)	27 (38.6%)	0.827
Do you know what a placebo is?	78 (51.0%)	45 (64.3%)	0.098
Do you know if you have the right to see the results of the study you are participating in?	48 (31.4%)	19 (27.1%)	0.507
Do you think your participation in the study will help other people in a similar future situation?	146 (95.4%)	69 (98.6%)	0.667
Do you know if you have obtained any benefits from participating in this study?	96 (62.7%)	46 (65.7%)	0.826

*Yes, or correct answer.

EI, educational intervention; Non-EIP, non-educational intervention patient.

A comparison of the answers of the surveys between the participants and the clinical research staff shows that, regarding the primary objective of the trial, Kappa is 0.599 (0.487–0.712)/0.658 (0.486–0.829) with no significant differences (*p* = 0.575). The use of placebo evidenced Kappa of 0.96 (0.89–1.00) in the NEIP group/0.80 (0.58–1.00) in the EIP group without finding any significant difference (*p* = 0.162). In relation to the extraordinary clinical visits, Kappa was 0.016 (−0.159–0.192)/0.056 (−0.099–0.217) without finding any significant difference (*p* = 0.721). Lastly, we compared the percentage of relative agreement regarding the meaning of study blindness and the result was 49 (46.2%)/20 (42.5%) without showing any significant difference (*p* = 0.806).

## Discussion

To the best of our knowledge, our study is the first to evaluate and improve the participants’ knowledge about their clinical trial, applying an educational intervention on medical research staff from multiple medical specialties. Patients’ knowledge about their involvement in clinical trials is limited. However, an educational intervention based on the fundamental values of bioethics and research clinical trials on the clinical research staff can enhance the process of empowerment and transit of information in the physician-patient relationship.

It should be noted that most patients included in clinical trials are men. This could be to some extent explained by a higher prevalence of disease in this gender, with an underrepresentation of women involvement in study trials, a concern already disclosed in guideline 18 of the International Ethical Guidelines for Health-related Research Involving Humans. Another interesting fact is the low educational level of the study population, since the National Institute of Educational Evaluation reports that 40% of the population between 25 and 65 years in Spain have elementary education. This could suggest a selection bias, complicating the extrapolation of the results of these studies to the general population.

The results show that a percentage of the patients were not aware of their inclusion in clinical trials, a percentage not affected by the educational intervention, which raises doubts about the validity of the ICF since there is a lack of patient inclusion awareness, even if a written ICF was signed. An inadequate transmission of the study information to the patient, resulting in unawareness of patient inclusion, could explain in part these results. In addition, the use of a very technical, unsuitable language for the education of the study population may also have contributed. So, according to this, subjects that are not able to understand their involvement in trials should not be included, or at least, if there is a sign of the patient willingness to participate, power should be given to their legal representation to make the decision to participate in clinical trials.

The answers concerning the willingness of the patient show an improvement after the EI, acquiring statistical significance on the topics of sufficient time to make the decision and the option given to the patient to take the ICF home to receive counseling and discuss it with friends and family or other physicians. This is particularly important to guarantee respect for the autonomy principle, and research is seen as a service to provide a better medical situation to others in the same situation and as a progress for the community.

The percentage of information items provided is increased after the EI, with a significant upgrading on the perception of the ability of patients to resolve their doubts and the understanding of the information provided, which results in a better understanding after the reading and verbal explanation of the ICF, according to the ICH. It depends solely on the investigators, so GCP training is essential and the process and correct timing of the ICF is crucial for the patient to make an appropriate decision.

Concerning the acknowledgment of the patient consent withdrawal, the results were similar to other studies (16), and increased after the EI. The acknowledgment of withdrawal is generally explained at the beginning of the ICF, but it is important to emphasize this concept and not to forget that the “research subject” is still a patient and not only a clinical subject. It is the function of investigators to encourage the patient at the decision-making time, all of this without affecting the physician-patient relationship. On the other hand, with regard to the number of patients who remember reading the entire ICF, although the literature shows better percentage outcomes (10), patient’s knowledge improved after EI. It must be noted that, while it is mandatory for the patient to read the entire ICF, there could be a social desirability bias predisposing patients to alter questionnaire answers as they perceive them to be desired by the investigator (17). Also our trial reported similar data compared to the literature with regard to good patient predisposition to participate in clinical trials (18), with rates above 90% which could be biased, since public health services are free in Spain. This could be because clinical research is seen as a service to improve a medical condition for others in the same situation, and as a health progress for the community.

One of the most significant aspects when a patient is invited to participate in a trial are the understanding of the risks and benefits that could be obtained in each study (19, 20). Compared to other studies, our subjects were more aware of the risks than of the benefits (16, 21). This marked difference can be explained because the investigators in these protocols may be emphasizing the benefits while neglecting the explanation of the risks, without having into account the implicit uncertainty of any clinical research trial.

Although there are no statistically significant differences arising after the EI, there is an improvement in the comprehension items. The range found is below the average, but similar to other studies, with regard to understanding of the concepts of study blindness and placebo, which every patient enrolled should know (10, 16). However, this can be explained by two reasons: the patients included in their respective protocols did not use these terms within the description of their trial, and comprehension may be influenced by the educational level and age (16, 22). It would be interesting to conduct future studies that focus on these aspects of patient knowledge. It could be interesting another joint reading of the ICF after its inclusion, reviewing key concepts would be helpful to fix this knowledge in the study. Also, a group educational intervention on the participants themselves could be interesting.

To be noted is the 24.2% subjects that delegate their decision to participate in trials to the investigator that obtained the ICF, which evidences the persistent idea of paternalism in our society, a practice that to date is described by other authors (17). This behavior must be rejected by the investigator; the patient should be empowered and must be reinforced to make their own health decisions considering the risks and benefits following the autonomy ethical value.

Another aspect to highlight is the poor knowledge of patients about the extraordinary scheduled visits and tests outside clinical practice; this can be aggravated by conducting research in public hospitals in the same services in which they provide standard care. But undoubtedly, it is an aspect to improve since some tests pose a serious risk, such as a coronary angiography, and their ignorance questions the understanding of their participation and therefore the validity of the ICF. On the other hand, more than half of the patients consider they have obtained benefits when participating in a clinical trial, perhaps motivated by those visits and tests outside the clinical practice that make the patient feel more protected and with a direct contact with the specialist in case of any eventuality (23).

The main limitation of our study is the failure to complete the total number of patients calculated to achieve conclusive results due to a redistribution of the Galician Regional Health Care System in between the inclusion period. Another potential limitation is the presence of the Hawthorne effect in the educational interventional group. These limitations do not imply any loss in the quality of our study results.

## Conclusion

In conclusion, patient understanding and knowledge of their involvement in clinical trials is limited in our study. However, an educational intervention based on the fundamental values of bioethics and research clinical trials made on the clinical research staff can improve the process of empowerment and transit of information in physician-patient relationship.

## Data availability statement

The data used to support the findings of this study are available from the corresponding author upon reasonable request.

## Ethics statement

This study has been approved by the Research Ethics Committee Recognized by Consellería de Sanidade, with registration number: 2014/329. Written informed consent for participation was not required for this study in accordance with the national legislation and the institutional requirements.

## Author contributions

PJ-S was the principal investigator and lead author. MM, VJ, CM, and AI were members of the initial research team and contributed to the development, editing, and writing of the manuscript. All authors met the International Committee of Medical Journal Editors requirements, read, and approved the last version of the manuscript.
