# Supplementary material for: Patients’ knowledge about their involvement in clinical trials. A non-randomized controlled trial
Source: Front Med (Lausanne). 2022 Sep 20;9:993086. doi: 10.3389/fmed.2022.993086 (PMC9531127; doi:10.3389/fmed.2022.993086)
Supplement: Supplementary file 1 [file Data_Sheet_1.pdf]

Nº 01/101

ANEX I: Questions to answer by the patient.

First of all, thank you very much for taking your time to answer this questionnaire that will be help to study the knowledge you, as the patients participating in this study, have. This study is promoted by the ________________________________. The principal investigator is ___________________ and you can contact him for any inquiries by calling the _____________, the unit's phone number. This questionnaire is **VOLUNTARY** and your refusal to participate will not affect in any way nor the relation between you and your physician neither the healthcare assistance you have the right to. It is also **ANONYMOUS** in the sense that no personal data will be asked. Thus, to guarantee both requirements you have to place the questionnaire once you have finished it in a specific box for this purpose. Before you do this, tear out the last sheet that should be empty to later pass it on to the physician or investigator that you have gone to see.

To answer the questionnaire, you should check one answer per question using an X over the chosen option.

1. Birth date: ________
2. Sex:
    a.☐Male
    b.☐Female
3. Marital status:
    a.☐ Single
    b.☐ Married or living with your couple
    c.☐ Divorced/Separated
    d.☐ Widow
4. Level of education:
    a.☐ Incomplete primary education
    b.☐ Primary education
    c.☐ Medium education
    d.☐ Higher education

5. Can you specify your employment status?

   a.☐ Employed

   b.☐ Unemployed

   c.☐ Housewife or househusband

   d.☐ Retired or pensioner

6. What language do you speak on a regular basis?

   a.☐ Spanish

   b.☐ Galician

   c.☐ Others: _______________

7. Are you participating in a research study?

   a.☐ Yes

   b.☐ No, I am not participating in any research study

IN THE CASE your answer is NO, you do not have to keep filling in this questionnaire. THANK YOU very much for your participation. You can now place it in the specific box of the study.

In the case that you have answered YES to the previous question, please, keep collaborating with us by answering the following questions:

1. Do you know the disease because of which you were invited to participate in the study?

   a.☐ Yes, because of (indicate) ______________________________________

   b.☐ No.

2. In which language was the informed consent form that was presented to you?

   a.☐ Spanish

   b.☐ Galician

3. Where have you been invited to participate in a research study?

   a.☐ In the consultation

   b.☐ During your hospitalization.

   c.☐ When you have been operated on.

4. The research study you participate in is related to:

   a.☐ Drug

   b.☐ Device.

   c.☐ Both.

   d.☐ None.

   e.☐ I do not know.

5. Have you been informed if you could get any benefits from your participation?
   a.☐ Yes, I have been informed
   b.☐ No, I am not aware of the possible benefits.
   c.☐ I know I will not get any benefits.
   d.☐ If I do not participate I could not receive the treatment.
6. Are you aware or have someone explained to you the risks of participating in the study?
   a.☐ Yes, I am aware of them.
   b.☐ No, no one has explained to me that there were risks involved.
7. Have you read the whole informed consent form before you accepted to participate in the study?
   a.☐ Yes
   b.☐ No
8. Do you think that you have had enough time to think through and give an answer according to your participation in the study?
   a.☐ Yes
   b.☐ No
9. Have you been offered the possibility to take it home and consult it with any relatives or friends before signing?
   a.☐ Yes
   b.☐ No
10. Do you think the written information that has been presented to you was enough?
   a.☐ Yes, enough
   b.☐ No, not enough.
   c.☐ Too much information and hard to understand.
   d.☐ Too much information but easy to understand.
11. Can you tell how many sheets has the informed consent form that you received?
   a.☐ Less than 20 sheets
   b.☐ Between 20 and 30 sheets.
   c.☐ More than 30 sheets
   d.☐ I have not received anything written.
   e.☐ I do not remember.
12. Have you got any copy of the informed consent form that you had to sign?
   a.☐ Yes, I have got a copy.
   b.☐ No, I have not got a copy.

13. Answer this question **ONLY** if you have checked **YES** in the previous one, If you received a copy of the informed consent form that you have signed, Is this copy signed by the investigator that invited you to participate too?

   a.☐ Yes, it was signed.

   b.☐ No, it was not signed.

14. Do you think that the information was easy to understand?

   a.☐ Yes, I have understood it easily.

   b.☐ Too much information but easy to understand.

   c.☐ Too much information and hard to understand.

15. Have you got the chance to talk with the investigator about the contents and any doubts you had?

   a.☐ No, I did not need any clarification, but I was offered the possibility.

   b.☐ I was offered the possibility and all my doubts were clarified.

   c.☐ I was not offered the possibility.

16. Are you satisfied with the clarifications that have been given to you according to the doubts that you could possibly had?

   a.☐ Not satisfied at all.

   b.☐ Mostly dissatisfied.

   c.☐ Somewhat satisfied.

   d.☐ Mostly satisfied.

   e.☐ Completely satisfied.

17. Are you aware of the reasons that lead the investigator to invite you to participate in the study?

   a.☐ You suffer from the disease that is being studied.

   b.☐ You suffer from the disease and you meet the criteria of the study.

   c.☐ The physician knows that I trust him and his or her criteria.

   d.☐ I do not know the reasons.

18. Do you think that you have understood the aim of the study you are participating in?

   a.☐ Yes

   b.☐ No

19. Do you know if you have any extraordinary clinical visits due to your participation in the study?

   a.☐ Yes, do you know how many? _____________

   b.☐ I do not have any more visits than the regular ones.

   c.☐ No, I do not know.

20. Do you know if you have any extraordinary tests due to your participation in the study such as ECGs, blood test, effort tests: ergometry, magnetic resonance, cardiac catheterization, etc.
    a.☐ Yes, I know that I will have to undergo some specific tests. Indicate____
    b.☐ No, I will not undergo any specific test for participating in the study.
    c.☐ I do not know.
21. Do you know what it means that your participation is blind?
    a.☐ Yes, it means that I will not know what treatment is given to me.
    b.☐ No, I do not know if my participation is blind or not.
    c.☐ No, I do not know the meaning of blind.
22. Do you know what it is a placebo?
    a.☐ Yes, it is a substance that has the same packaging of a drug but that does not contain any drug.
    b.☐ Yes, it is a substance that has a different packaging but that contains the same drug.
    c.☐ No, I do not know what it means.
23. Do you know if placebo is used in the study that you are participating in?
    a.☐ Yes, placebo is used in this study.
    b.☐ Placebo is not used in this study.
    c.☐ No, I do not know.
24. Can you withdraw the informed consent form and leave the study whenever you want?
    a.☐ I cannot withdraw.
    b.☐ I can withdraw whenever I decide to without having to give any sort of explanations but it is suggested to inform the investigator.
    c.☐ I can only withdraw the study if I have experienced any adverse event.
25. Do you know where this study is been carried out?
    a.☐ Only in this hospital.
    b.☐ In other hospitals in Spain.
    c.☐ In other countries as well.
    d.☐ I do not know.

26. Do you know if you have obtained any benefits for participating in this study?

a.☐ Yes, I consider that I have obtained a better clinical assistance and a better follow-up of my disease.

b.☐ No, I do not consider that I have obtained any benefits.

c.☐ I do not know.

27. Have you ever participated in any other research study before this one?

a.☐ Yes

b.☐ No

28. Would you like to see the results obtained from the research you are participating in?

a.☐ Yes

b.☐ No

29. Do you know if you have the right to see the results of the study you are participating in?

a.☐ Yes

b.☐ No, I do not have the right to see the results of the study.

c.☐ I do not know.

30. Do you think that your participation in the study will help other people in a similar future situation?

a.☐ Yes

b.☐ No

31. Would you mind to participate in a future study if you were asked to?

a.☐ Yes

b.☐ No

Thank you very much for having the questionnaire completed. Now you have to steer out the following sheet, ANEX II, to pass it on to the physician or investigator that you have seen. First, to guarantee the anonymity of your answers you have to introduce this questionnaire in the specific box for this purpose.

ANEX II: *Questions to complete by the investigator:*

This questionnaire is ANONYMOUS, and no personal data will be gathered. As a guarantee, you will be asked to introduce the questionnaire in a specific box when completed.

GENERAL ASPECTS

1. Unit of the study: ___________________
2. Date of inclusion: ____________________
3. The aim of the study is a:
   - **a.**☐ Device:
   - **b.**☐ Drug:
   - **c.**☐ Both:
   - **d.**☐ None:
4. Number of pages in the informed consent form: _____ (include any additional sub-studies).
5. Number of follow-up visits for participating in the study: ______
6. This study IMPLIES (choose one of the options):
   - **a.**☐ Undergoing specific tests (that will not be carried out in regular clinical practice), in this case, indicate which tests ___________________________
   - **b.**☐ It only implies data gathering during regular clinical practice.

SOME ASPECTS RELATED TO THE DESIGN OF THE STUDY

7. If it is a clinical trial involving drugs, indicate the phase: **I, II, III, IV**
8. Multicenter: YES/NO
9. International: YES/NO
10. Comparator studies (choose one):
    - **a.**☐ SIMPLE BLIND
    - **b.**☐ DOUBLE-BLIND
    - **c.**☐ OPEN (not BLIND)
11. Randomization (choose one):
    - **a.**☐ RANDOMIZED
    - **b.**☐ NOM RANDOMIZED
12. Placebo-controlled: YES/NO
